# Factors associated with adolescent school girl’s pregnancy in Kumbo East Health District North West region Cameroon

**DOI:** 10.11604/pamj.2018.31.138.16888

**Published:** 2018-10-25

**Authors:** Layu Donatus, Dohbit Julius Sama, Joyce Mahlako Tsoka-Gwegweni, Samuel Nambile Cumber

**Affiliations:** 1 Department of Reproductive Health, Faculty of Health Sciences, Catholic University of Central Africa, Yaoundé, Cameroon; 2 Faculty of Health Sciences, University of the Free State, Bloemfontein, South Africa; 3 Section for Epidemiology and Social Medicine, Department of Public Health, Institute of Medicine, The Sahlgrenska Academy at University of Gothenburg, Gothenburg, Sweden

**Keywords:** Adolescents, factors, pregnancy, health district

## Abstract

**Introduction:**

Teenage pregnancy is a social problem in Cameroon in general and in Kumbo East in particular. This results in physical, psychological and socio-economic consequences on the teenage mother, family and the society as a whole. In spite of studies and interventions that have been and are being implemented, the prevalence of unplanned teenage pregnancy in Kumbo East Health District is still high, suggesting that more efforts are required to achieve effective preventive measures. The aim of this study was to determine factors associated with adolescent school girl's pregnancy in Kumbo East health district.

**Methods:**

A cross-sectional descriptive study design was used and a simple random sampling technique was used to select 293 respondents aged 15 to 19year. The district hospital antenatal clinics and the Health Centres were selected. Data was obtained from 292 participants under the age of 20 years who were willing using a questionnaire administered through face-to-face interviews.

**Results:**

The study show a high prevalence (60.75%) of teenage pregnancy in the sampled antenatal clinics of Kumbo East Health District attributable to inadequate considerations given to factors associated with school girl's pregnancy. This study has indicated that the age of teenager at first pregnancy, low contraceptive use, socio-economic status and physical violence are factors that are greatly associated with teenage pregnancy. Among the reasons contributing to the low use of contraceptives are: sexually activity, lack of knowledge, fear of side effects, including sterility, condoms disappearing in the womb and inequality of power with sexual partners. This study shows that teenagers obtain information mainly from school (53%) and relatives (20%).

**Conclusion:**

The use of contraceptive alone may not reduce teenage pregnancy, however double method is very effective but addressing the impact of poverty on teenagers, empowering them on their rights and information in order to make right choices is very important.

## Introduction

Teenage pregnancy is considered as one occurring in a young woman who has not reached her 20th birthday. This definition is applicable irrespective of the legal status of the marriage of the woman or legal age to consider an individual as adult [1]. 16 million girls aged 15-19 years give birth each year, most prevalent in low and middle-income countries located in sub-Saharan Africa [1]. In the developing world, one-third to one-half of women become mothers before the age of 20 and pregnancy related complications have become the leading causes of death among them [1, 2]. Teenage pregnancies are a global phenomenon. The pregnancy rate among teenagers in USA was 6.78% of pregnancies per 1,000 women aged 15-19 in 2008. [3]. Among the countries in the Western Europe, the United Kingdom [UK] has the highest teenage conception and abortion rates [2, 4]. The report presents an update on the current situation of pregnancies among girls less than 18 years of age and adolescents 15-19 years of age; trends during the last 10 years; variations across geographic, cultural and economic settings; interventions available to minimize pregnancy among adolescents; evidence for these programmatic approaches; and challenges that nations will have to deal with in the next 20 years given current population momentum [5]. The concentration of adolescent girls aged 10 to 17 will also change significantly, with the largest increase occurring in sub-Saharan Africa, where adolescent pregnancy is most common, and the rate of contraceptive use the lowest in the world [5]. A study conducted in Malawi showed that 57% of teenage girls opt to risk pregnancy rather than asking a partner to use a condom [2]. In Malawi, there is a high prevalence of casual sex among teenagers who shun condoms although they engage in multiple relationships. Scholars in the field argue that, because of the risk associated with high prevalence of early sexual behaviour, low contraceptive use, and many early pregnancies, adolescents in Cameroon are an important target group for sexual and reproductive health programs [6, 7]. In order to prevent early age pregnancies, it is important to make sure that adolescents have the means to make informed and healthy choices concerning their sexual and reproductive health. Yet, as it stands, reproductive health and family planning services in Cameroon mainly target older married women, and adolescents often remain largely overlooked [5].

Adolescent school girl's pregnancies vary from country to country and in Cameroon, reproductive health remains a major public health challenge with elevated maternal mortality rate. The maternal mortality rate is estimated at 782 deaths per 100,000 live births [8]. This high mortality rate remains a dilemma because it involves the young mothers at the moment where they are giving birth. In addition, adolescent contribute 28% of maternal mortality in Cameroon [8]. According to DHS 2011, 25.6% of adolescents 15 to 19 have started sexual intercourse and 21% of then have had a child and 4% are pregnant for their first pregnancy. Still from the same source fertility rate of this age group is 127‰ which increase rapidly and attained a maximum of 250‰ within the age group 25-29yrs, suggesting that teenage pregnancy may be on the increase. The proportion of adolescents who have started fertility grow rapidly with age, 5% for 15yrs to 48% for 19yrs and the rate stood at 18% in the north west region [8]. Therefore, reducing teenage pregnancy, chiefly by promoting the use of contraceptives, will be necessary in order to prevent consequences that are associated with teenage pregnancy. Nevertheless, lessons from Zimbabwe and South Africa suggest that promoting the use of contraceptives alone does not necessarily reduce teenage pregnancy in developing as in developed countries. Therefore, other factors may be playing a major role as mentioned earlier. The general objective was to determine factors associated with adolescent school girl's pregnancy in Kumbo East Health District.

## Methods

A crossectional descriptive study was selected because it incorporates a descriptive component that enables the calculation of the prevalence of teenage pregnancy in antenatal clinics of KEHD. The socio-economic status where measured by asking respondents questions concerning their househood items they possessed; bicycle, car , family income were collected and the knowledge was assessed by structured questions to evaluate the knowledge of respondents to factors associated with teenage pregnancy. The questionnaire was adopted from WHO standard guide and from the research of other researchers. The questionnaire was then pre-tested and adjusted to fit the context. Four midwives' students who were in their final year and had some experience in data collection assisted in the study. They were given orientation on the process for data collection and management prior to the commencement of field studies. They were trained for a period of three days in the administering of the questionnaire and research ethics, with an emphasis on informed consent. Each research assistant was given a code that was used for identification in case of any queries. Data was then collected entered into the computer and stored in external hard drive and USB keys. Data was then assembling and the analysis started. Our research period was for 10 months with the study population consisting of all school girl's pregnant aged [15-19yrs] from Kumbo East Health District. The sample size were then pregnant teens (15 to 19yrs) attending antenatal clinics in selected health centres in Kumbo East Health District at the time of data collection. Antenatal and infant welfare clinics were used in order to access pregnant adolescents.

**Inclusion criteria:** All school teens age 15 to 19 yrs who are pregnant who attend: ANC consultation irrespective of their trimester at SEGH and selected health centre, infant welfare clinics, and who give their consent.

**Exclusion criteria:** Pregnant teens who came to the clinic and didn't give their consent were excluded. Although teenage pregnancy concerns both sexes, male teenagers were excluded as they rarely attend antenatal services with their partners.

**Sample method and sample type:** The sampling method used was a simple random technique; the names of the health centres within was written on a piece of paper and placed in a container, four names were picked at random from the container. Jakiri integrated health centre, sub-divisional hospital Jakiri, St. Jude clinic and Shisong General Hospital. Our sample type was a convenience sampling. The Kumbo East District Hospital [Shisong] represents urban teenage mothers who may have experienced different factors from the rural setting. To obtain an adequate sample, all pregnant women visiting the antenatal clinics and infant welfare clinics over a three-month period at the selected sites under the age of 20 were interviewed.

**Sample size in a cross sectional study:** To calculate our sample size, we are going to use the Lorenz formula which stipulates, P= Prevalence of adolescent 15 to 19 (p = 0.25), t = confidence level [95%= 1.96], e = error margin [0.05], N = Sample size.

$$A={\left[t\right]}^{2}*p[1-p]{/}_{e2}$$

Statistical application of this formula, resulted to a sample size of 293, implying that the study will recruit a total of 293 participants from the selected hospitals.

**Data collection technique:** Data was collected over a period of 3 months from August to October 2017, because of the Anglophone crisis with numerous “ghost towns” that reduce Antennal consultation by pregnant women difficult and hence leading to a long period of time to collect data. Using a standardized questionnaire administered through face-to-face interviews. The Questionnaire consisted mostly of closed questions. Each questionnaire took 20-40 minutes to administer. There were no refusals - all participants willingly consented to participate in the study. The arrangements for confidentiality and privacy ensured that respondents accepted to be interviewed and spoke more freely on the subject. In addition, it was also possible that respondents felt that there was somebody to listen to their problems. The face-to-face interviews facilitated responses and the quality of information and this method was convenient since most of the participants had low literacy levels.

**Statistical consideration:** Data collected were entered into the CDC-Epi-Info version 7.2.2.2, transferred to MS-Excel and then exported to SPSS version 21.0 software. Data cleaning was performed with MS-Excel to check for inconsistencies in data entry and responses, prior to analyses. Associations between planed [Yes/ No] pregnancy and various variables were evaluated using Pearson and Yate's chi square [φ^2^] tests. Measures of association; OR and Pearson's chi square [φ^2^] tests were calculated by use of CDC-Epi-info version 7.2.2.2 and SPSS version 21.0 for the establishment of associations or differences (un) planed pregnancy and various variables.

**Ethical considerations:** in order to protect the rights of the interviewees and meet requirements for research involving people, clearances were obtained from authorities and informed consent from the participants. The authorization was also obtained from the North West Regional Delegation of Health. Ethical clearances were obtained from the Committee for Research on Human Subjects of the Catholic University of Central Africa (UCAC). Clearances to use health facilities were also obtained from the Kumbo East District Health Office. The following points were clarified: participation in the study was voluntary; participants were free to withdraw at any time without coercion and there were neither direct benefits nor known risks at any time. To ensure anonymity and privacy, numbers were used instead of names on the questionnaires. Interviews were conducted in private rooms which were provided at the clinics for this purpose. The completed questionnaires that were not entered to the computer were locked in a cupboard accessible to the researcher only to ensure confidentiality.

## Results

**Socio-demographic characteristics:** A total of 293 participants of average age 18.88yrs were sampled from 13 shortlisted localities of KEHD; Jakiri had the highest proportion 65 [22.18%] of participants while Noi and Tan had the lowest 11 [3.75%] each. Out of the 293 participants, 154 [52.56%] admitted currently schooling. Out of the 293 ladies sampled, 153 (52.22%) were single. Out of 293 sampled in the study, 175 [59.73%] who had not delivered, while the remainder had delivered at least a child, either in marriage or out of marriage. Socio-demographic data was collected for variables such age, marital status, alcohol and drug/substance attitudes, as well as information concerning partner (Table 1).

**Table 1:** Demographics; age, religion, school attendance and parity

Variable	Attribute	Frequency	Percent (%)
**Age groups (years)**	15 - < 20	227	77.47
	20 - < 25	48	16.38
	25 - < 27	2	0.68
	≥ 27	16	5.46
	**Total**	**293**	**100.00**
**Quarter of residence**	Jakiri	65	22.18
	Kouwong	21	7.17
	Kumbo	19	6.48
	Mantum	26	8.87
	Ngoilum	20	6.83
	Nkar	17	5.80
	Noi	11	3.75
	Nsom	29	9.90
	Ntutiy	14	4.78
	Shisong	23	7.85
	Shiy	20	6.83
	Tan	11	3.75
	Waitakwar	17	5.80
	**Total**	**293**	**100.00**
**Religion**	ATR*	39	13.31
	Catholic	95	32.42
	Muslim	74	25.26
	Protestant	85	29.01
	**Total**	**293**	**100.00**
**Currently schooling**	Yes	154	52.56
	No	139	47.44
	**Total**	**293**	**100.00**
**Parity**	None	175	59.73
	One	95	32.42
	Two	2	0.68
	Three	18	6.14
	Four	3	1.02
	**Total**	**293**	**100.00**

* ATR = African Traditional Religion

**Sources of information on sexual and reproductive health:** Despite the diversity of the sources of information on a variety of issues, 20.48% and 2.05% claimed ignorance of information on sex and sexuality as well as the use of contraceptives. However, a majority of the participants; 79.52% and 97.95% had at least one of the variety of sources of information on sex and sexuality and use of contraceptives. A majority of the participants had learned of sex and sexuality as well as use of contraceptives from school; 38.20% and 52.90%, respectively. Only 13.31%, of mothers educated their daughters on sex and sexuality. Books, Counsellors, local radio and peers respectively served as 6.83%, 19.80%, 13.65% and 20.48%, sources of information on sex and sexuality to adolescents. Out of the 293 respondents, 11.60%, 13.31%, and 20.14% had learned of contraceptives from friends, hospital, and relatives respectively (Table 2).

**Table 2:** Sources of information on SRH and contraceptive use

Source(s) of information	Attribute	Frequency	Percent
On sex & sexuality*	Books	20	6.83
	Mother	39	13.31
	Counsellor	58	19.80
	School	134	38.20
	Radio	40	13.65
	Peers	60	20.48
	None of these	60	20.48
On use of contraceptives*	Friends	34	11.60
	Hospital	39	13.31
	Relatives	59	20.14
	School	155	52.90
	None of the above	6	2.05
	**Total**	**293**	**100.00**

* Dichotomy group tabulated at value 2

**Knowledge of information on sexual and reproductive health (SRH):** Considering the respondents answers to question ask on whether their menstrual and reproductive characteristics , their knowledge of information on sexual and reproductive health (SRH) was assessed, a majority of the respondents; 66.2%, 66.6%, 59.7% and 93.2% out rightly admitted that it is true that; amenorrhoea leads to accumulation of dirt and sickness, disappearance of a condom into a woman during sex, most pregnancies occur in the middle of the menstrual cycle as well as, one can become pregnant during the first sexual intercourse. Another majority; 93.2%, 53.2%, 65.9% and 53.6% actually admitted as outright false the fact that; frequent sex prevents pregnancy, sterility arises from the use of contraceptive use, washing of genitals after sex prevents pregnancy and the consumption of sachet whisky prevents conception (Table 3).

**Table 3:** Knowledge of information on sexual and reproductive health (SRH)

Knowledge	PT (%)	T (%)	PF (%)	F (%)
Most common cause of infertility is STI	40 (13.7)	77 (26.3)	38 (13)	138 (47.1)
Sex during menstruation can lead to pregnancy	58 (19.8)	19 (27.0)	20 (6.8)	136 (46.4)
A woman can become sterile upon using depo	78 (26.6)	117 (39.9)	20 (6.8)	78 (26.6)
Amenorrhoea leads to accumulation of dirt and sickness	59 (20.1)	194 (66.2)	0 (0.0)	40 (13.7)
Intercourse in water cannot prevent pregnancy	19 (6.5)	116 (39.6)	60 (20.5)	98 (33.4)
Condom usage can disappear into the woman	58 (19.8)	195 (66.6)	0 (0.0)	40 (13.7)
Frequent sex prevents pregnancy	0 (0.0)	0 (0.0)	20 (6.8)	273 (93.2)
Use of pills after pregnancy can lead to abortion	39 (13.3)	58 (19.8)	60 (20.5)	136 (46.4)
The use of contraceptives is promiscuity	39 (13.3)	60 (20.5)	58 (19.8)	136 (46.4)
Most pregnancy occurs in the middle of the cycle	19 (6.5)	175 (59.7)	0 (0.0)	99 (33.8)
No pregnancy in 4 months after contraception is sterility	0 (0.0)	97 (33.1)	40 (13.7)	156 (53.2)
One can be pregnant at first sex	20 (6.8)	273 (93.2)	0 (0.0)	0 (0.0)
Washing genitals after sex prevents pregnancy	40 (13.7)	40 (13.7)	20 (6.8)	193 (65.9)
Sachet whisky consumption prevents pregnancy	58 (19.8)	78 (26.6)	0 (0.0)	157 (53.6)

**Factors associated with unplanned teenage pregnancy in KEHD:** A list of factors were provided in the questionnaire for the respondent to tick and out of the 293 respondents, 61.09% claimed never to have used the condom, and 52.56% said their partner was not the father of their child. 46.42% of the 293 participants said they do not use contraceptives, 13.65% failed to disclose the method of contraceptive used, by simply saying “none of the above”, when asked, when they started using contraceptive, 58.02%, said they have never used contraceptives (Table 4).

**Table 4:** Factors associated with adolescent school girl’s pregnancy

*Non use of contraceptives*		
Have you ever used condom?	Frequency	Percent (%)
Yes	114	38.91
No	179	61.09
Total	293	100.00
**Is partner father of this child?**		
Yes	139	47.44
No	154	52.56
Total	293	100.00
**Contraception and method**		
Condom	44	15.02
I don't use contraceptives	136	46.42
Implant	15	5.12
IUCD	24	8.19
Pill	34	11.60
None of the above	40	13.65
Total	293	100.00
**When did you first use contraceptives?**		
This year/ 2017	6	2.05
1 year ago/ 2016	71	24.23
2 years ago/ 2015	22	7.51
3 years ago/ 2014	8	2.73
4 years ago/ 2013	8	2.73
5 years ago/ 2012	8	2.73
Never	170	58.02
Total	**293**	**100.00**
**Sources of knowledge of contraceptive**		
Friends	34	11.60
Hospital	39	13.31
Relatives	59	20.14
School	155	52.90
None of these sources	6	2.05
**Why I have never used contraceptives**		
I am scared of nurses at the clinic	20	6.83
I was scared my parents would find out	59	20.14
It is against my religion	20	6.83
My boyfriend/ partner did not want me to	38	12.97
None of these	156	53.24
**Total**	**293**	**100.00**

**Lack of knowledge on sexual reproductive health, circumstances of first sex, physical, sexual and substance abuse:** From the answers provided by the respondent concerning knowledge assessment, 20.48% of the respondents had information on sex and reproductive health from their peers and, another 20.8% could not really identify their source of information on sex and reproductive health. 11.60% and 2.05% of the respondents learned of how to use contraceptives from friends and no defined sources (Table 5).

**Table 5:** Circumstances of first sex, first sexual experience, relationship control and duration of relation

Why you relate to partner	Frequency	Percentages (%)
Have A Good Time	38	12.97
Be Married	40	13.65
Be Provided With Clothes	19	6.48
Be Provided With Cosmetics	0	0
Be Provided With Food	0	0
Be Provided With Money	0	0
**Drug/ substance use by partner**		
Marijuana	11	3.75
He doesn't use drugs	197	67.24
Any other drug	44	15.02
Any injectable drug	19	6.48
**Drug/ substance use by me**		
Any Other Drug	38	12.97
I don't use drugs	236	80.55
Marijuana	19	6.48
**Total**	**293**	**100.00**
**First sexual experience**		
I was persuaded	136	46.42
I was raped	19	6.48
I was tricked	64	21.84
I was willing	22	7.51
None of the above	52	17.75
**Total**	**293**	**100.00**

**Characteristics of sexual partner and gender power relations in partnership:** The sexual partners of respondents were of the age range 16-32 years (mean age of 22.815). At their age, a majority 67.24% of the respondents claimed their partners do not use drugs, while 6.49%, 15.02% and 3.75% respectively claimed their partners; do any injectable drugs, any other drugs and marijuana. On alcohol consumption frequency; 49.83% claimed not to know the drinking rate of their spouses, 7.85%, 20.82% and 21.50% said their partners drink at the rate of; only at weekend, less than once a month and a few times a month respectively (Table 6).

**Table 6:** Alcohol/ drug attitude of partner

Partners drinking frequency	Frequency	Percent
A few times in a month	63	21.50
I don’t Know	146	49.83
Less than once a month	61	20.82
Only at weekends	23	7.85
**Total**	**293**	**100.00**
**Drug/ substance**		
Marijuana	11	3.75
Any other drug	44	15.02
Any injectable drug	19	6.48
He doesn't use drugs	197	67.24

## Discussion

### General patterns of teenage pregnancy in Kumbo East Health District

The prevalence of teenage pregnancy in Cameroon is 28% and in Kumbo East Health District the results on this study shows that the prevalence of unplanned teenage pregnancy is 60.75% which very high this could be attributed to the fact that schools were not operational at the time of this study due to political unrest in the country at the time of data collection. Out of the 293 respondents, 61.09% claimed never to have used the condom, and 52.56% said their partner was not the father of their child. 46.42% of the 293 participants said they do not use contraceptives, 13.65% failed to disclose the method of contraceptive used, by simply saying “none of the above”, when asked, when they started using contraceptive, 58.02% said they have never used contraceptives. This implies that there is still a need for more efforts. Possible factors contributing to the high prevalence in this study area include age at first pregnancy, low contraceptive use, educational levels and socio-economic status, circumstances at first sex, lack of knowledge on reproductive and sexual health and physical and sexual violence.

### Knowledge and sources of information on reproductive and sexual health

Considering the respondents answers to question ask on whether their menstrual and reproductive characteristics , their knowledge of information on sexual and reproductive health [SRH], a majority of the respondents; 66.2%, 66.6%, 59.7% and 93.2% out rightly admitted that it is true that; amenorrhoea leads to accumulation of dirt and sickness, disappearance of a condom into a woman during sex, most pregnancies occur in the middle of the menstrual cycle as well as, one can become pregnant during the first sexual intercourse. Another majority, 93.2%, 53.2%, 65.9% and 53.6% actually admitted as outright false the fact that; frequent sex prevents pregnancy, sterility arises from the use of contraceptive use, washing of genitals after sex prevents pregnancy and the consumption of sachet whisky prevents conception (Table 4).

### Age of the teenagers

The ages of participants ranged from 15-27 years, with mean age 18.88 years and modal age group of 15 -< 20 years with 227 [< 77.41%]. A large majority of participants 59.73% had no child, while 32.42% had one child, 0.68% had two children, 6.14% had three children and 1.02% had four children. In Cameroon, Kenya, South Africa and Canada, the average age difference between teenagers and their sexual partners is 15, 7, 5 and 2.6 years, respectively. In addition, teenagers who marry older men often have less power in decision making around sexual intercourse, childbearing and the use of contraceptive [1].

### Knowledge contraceptive use

On the usage of contraceptives, 15.02% used the condom, 5.12% claim to use implants, 8.19% claim to use IUCD, while 11.60% use pills. On when they began using contraceptives; 2.05% began this year, 24.23% began last year, 7.51% began in the year 2015, 2.73% began in 2014 and 2013 as well as 2012 (Table 4). Respondents also advanced possible fears and reasons for not using contraceptive; 6.83, were scared of nurses, 20.14% were scared of their parents, 6.83% claimed it was against their religion and 12.97% admitted their partners were not in favour of it (Table 1). The fears, beliefs and perceptions of sexual inactivity associated with contraceptive use may be attributed to the lack of knowledge and low levels of awareness. This is conformity with a similar study Contraceptive prevalence in Cameroon is low among married women and shows uneven distribution varying from 2.6% in North Cameroon to 43.9% in Yaoundé [6, 7]. The low prevalence use among married couples suggests that once a woman is married she is exposed to pregnancy irrespective of her age. In Cameroon, unmet needs for family planning are 22%. And the use of modern contraception in Cameroon is about 16%. Reasons for non-contraceptive use include, religious and cultural beliefs, poor quality of services, including the negative attitude of service providers, fear of exposure of their bodies, having adults at the same services and inability to negotiate contraceptive use with sexual partners. Furthermore, misconceptions, fear of side effects and stigma associated with the use of contraceptives as adolescents may be labelled as being promiscuous can also be considered as contributing factors for non-contraceptive use [1]. Similarly, in South Africa, a study revealed that teenage pregnancy is attributed to the low utilization of contraceptives, especially on their first intercourse, due to the lack of access to medical information on reproductive system, inaccessible family planning services, gender inequality and decision on the social life, fears about contraception on fertility and menstruation and condoms could be left inside the vagina or womb [4]. It was therefore envisaged that the use of contraceptives among teenagers in the study area could be low, making it a factor for pregnancy.

### Levels of education, cultural practices and economic factor

The ages of the partners of participants ranged from 16-32 years; Half, 50.17% of partners of adolescent school girls were in the age group 20 - < 25 years. 17.41% were in the age group 16 -< 20 years, 25.26% in the group 25 -< 30 years and 7.17% in the age group 30 - 32 years. With 51.88% of the 293 participant's partners had secondary school education, followed by those with tertiary education 40.27%, those with no formal education were 6.83% and primary education were 1.02%. Of the 293 participants, the partners of 44.37%, 40.96% and 14.68% were respectively studying, unemployed and working (Table 2). The study agrees that Teenage girls may indulge in sexual activity in exchange for goods, money and experiences such as taking meals in hotels [1]. A study in Malawi, found that 66% of adolescents had accepted money or gifts in exchange for sex and in some cases, parents may encourage their daughters into relationships with men for consumer goods or a girl may go out with men because her parents cannot give her the basic needs. Teenagers with unplanned pregnancy are more likely to come from low socio-economic status than with planned pregnancy [1]. The level of education of parents, especially the mother, may have an influence on the adolescent towards teenage pregnancy as she acts as a role model [9] which may be a preventive factor of teenage pregnancy. In Kenya it was reported that women with no education had first sexual intercourse three years earlier than their counterparts with at least a secondary school education [1]. The low literacy levels may lead to low paying jobs, causing early marriage and influencing non-contraceptive use, thereby increasing the prevalence of teenage pregnancy. Therefore, it was expected in the study area that the low literacy levels may have an impact on unplanned teenage pregnancy. Culturally, amongst factors associated with unplanned teenage pregnancy were, age at onset of menstruation, age at first sex, who they live with as well as the marital status of their parents. It was difficult to prove that culture is a factor. Economically, these were age, religious and delivery status of participants (Table 1), marital status of participants and parents, partner's educational and occupational status.

### Gender power relations in partnerships

There were a variety of circumstances that could lead to sex; partner violence, reasons for involvement in a relationship and first sexual experience. For partner violence; 19.80%, had their funds managed by their partners, 33.11% were forced by their partners into sex, 20.14% would be hit by their partners and 40.27% had their funds managed by their partners. 6.48%, 13.65% and 12.97% of respondents respectively got involved in a relationship to be provided with clothes, to be married and to have a good time. For the first sexual experience; 46.42% were persuaded either by a relation or boyfriend, 6.48% were raped, 21.84% were raped, 7.51% were willing and collaborated or 17.75% refused to disclose their first sexual experience (Table 5). This means that male partner is the one who decides what happen in a relationship.

### Lack of knowledge on sexual reproductive health, circumstances of first sex, physical, sexual and substance abuse

20.48% of the respondents had information on sex and reproductive health from their peers and, another 20.8% could not really identify their source of information on sex and reproductive health. 11.60% and 2.05% of the respondents learned of how to use contraceptives from friends and no defined sources (Table 3). Most studies conducted in developing countries report that adolescent girls often lack basic knowledge about reproductive and sexual health [1]. This study contrast with that of findings of Nanze. C.C (2006 on risk factors of unwanted/uplanned pregnancy in zomba district, Malawi) indicate that communication about reproductive and sexual matters within families is limited, forcing girls to get information chiefly from peers, boyfriends and teachers. On drug and substance use, a wide majority (80.55%, said they do not take drugs 12.97% said they took any drug, while 6.48% admitted smoking “banga” (Table 7). Similar findings have been reported in South Africa [9]. Unlike wise in Cameroon, it is not common for teenage girls to use drug substances due to cultural values. However, there may have been underreporting since drug use is illegal, making it a sensitive topic.

**Table 7:** Circumstances of first sex, first sexual experience, relationship control and duration of relation

Why you relate to partner	Frequency	Percentages (%)
Have A Good Time	38	12.97
Be Married	40	13.65
Be Provided With Clothes	19	6.48
Be Provided With Cosmetics	0	0
Be Provided With Food	0	0
Be Provided With Money	0	0
**Drug/ substance use by partner**		
Marijuana	11	3.75
He doesn't use drugs	197	67.24
Any other drug	44	15.02
Any injectable drug	19	6.48
**Drug/ substance use by me**		
Any Other Drug	38	12.97
I don't use drugs	236	80.55
Marijuana	19	6.48
**Total**	**293**	**100.00**
**First sexual experience**		
I was persuaded	136	46.42
I was raped	19	6.48
I was tricked	64	21.84
I was willing	22	7.51
None of the above	52	17.75
**Total**	**293**	**100.00**

## Conclusion

The study factors associated with adolescent school girl's pregnancy in KEHD was aimed at examining the factors associated with teens pregnancy. A descriptive study was used and the teens 15 to 19yrs were selected. Teenage pregnancy whether planned or unplanned is detrimental to the health and socio economic status of the teenagers. This study shows that there is a high prevalence (60.75%) of teenage pregnancy in the sampled antenatal clinics of Kumbo East Health District attributable to the low contraceptive use and socio-economic status, lack of reproductive and sexual knowledge, circumstances at first sex, including force and rape, physical and sexual violence, early marriage, age of sexual partner and alcohol abuse. Among reasons contributing to the low use of contraceptives are; lack of knowledge, fear of side effects, including sterility, condoms disappearing in the womb and inequality of power with sexual partners. Teenagers obtain information mainly from school (53%) and relatives (20%). The high prevalence of unplanned teenage pregnancy, low contraceptive use, myths and misconceptions surrounding the use of contraceptives indicate that teenagers are receiving inaccurate information about reproductive and sexual health, a problem which may be compounded by low educational levels. Therefore, the lack of knowledge about reproductive health is a factor for teenage pregnancy. The lack of basic necessities is one of the factors that force teenagers to engage in transactional sex. Based on the study it is clear that teenagers have no control over sexual and reproductive health since their sexual partners are the sore decision makers making physical and sexual violence a factor for teenage pregnancy. However, the influence of cultural practices and religion, substance abuse, relationship control, and fear of parents and intimidating attitude of health service providers have been supported as factors. The associations suggested by the study point towards a need for greater emphasis in reproduction and sexual health promotion interventions. In addition, strengthening awareness and giving information to dispel fears, misconceptions and rumours about contraceptive use will prepare teenagers for the physical changes they might experience when adopting contraceptive methods. Nevertheless, contraceptive use alone may not reduce teenage pregnancy, but addressing the impact of poverty on teenagers, empowering them on their rights and information in order to make right choices is very important.

**Recommendations:** The study recognizes efforts being made by government and non-governmental organizations to solve the problem of teenage pregnancy in north west region and Kumbo East in particular. These efforts include promoting the use of contraceptives, education of girls and poverty alleviation. However, the low use of contraceptives and socio-economic status, continued sexual and physical violence, the feeling of inferiority due to age differences with sexual partners and early marriages indicate that factors for teenage pregnancy still persist in the study area. Therefore, there is a need to develop programmes to address factors identified in this study. Solutions to the problem require multidisciplinary implementing teams, including parents, schools, communities, NGOs and government sectors. The following recommendations are suggested.

### What is known about this topic

- Knowledge of this geographic distribution can provide useful information for updating and strengthening adolescent reproductive health strategies in Cameroon;
- In Cameroon, the prevalence of adolescent pregnancy is between 40% to 50%;
- Adolescent age 15 to 19 contribute to 28% of maternal mortality in Cameroon.

### What this study adds

- Study will help provide few answers to this problem and may be prick health leaders to react towards the amelioration of some of their health strategies concerning reproductive health especially adolescent health in rural areas of Cameroon;
- Moreover, it will also go a long way to convince national and international partners to continue to largely invest in the reproductive health section;
- This will stand as a pillar to help adolescent to adopt responsible behaviours in regard to adolescent pregnancy especially at individual, community and health systems levels.

## Competing interests

Authors declare no competing interests.

## References

[1] Chen XK, Wen SW, Fleming N, Demissie K, Rhoads GG, Walker M (2007). Teenage pregnancy and adverse birth outcomes: a large population based retrospective cohort study. Int J Epidemiol.

[2] UNICEF (2008). Fact Sheet: World Population Day.

[3] Kathryn Kost and Stanley Henshaw (2008). U.S. Teenage Pregnancies, Births and Abortions, 2008: National Trends by Age, Race and Ethnicity.

[4] Kaphagawani Nanzen Caroline Chinguwo (2008). Risk factors for unwanted / unplanned teenage pregnancy in Zomba District, Malawi.

[5] DHS (2011). Cameroon demographic health survey.

[6] UNFPA (2013). Adolescent pregnancy: a review of the evidence.

[7] Tebeu PM, Kemfang JD, Sandjong DI, Kongnyuy E, Halle G, Doh AS (2010). Geographic Distribution of Childbirth among Adolescents in Cameroon from 2003 to 2005.

[8] Demographic Health Survey statistics (2009). Health data and indices.

[9] Vundule C, Maforah F, Jewkes R, Jordaan E (2001). Risk Factors for Teenage Pregnancy among Sexually Active Black Adolescents in Cape Town. South African Medical Journal.

